# Two Case Reports of Intravenous Leiomyomatosis with Hyaluronan Expression

**DOI:** 10.1155/2018/4039183

**Published:** 2018-07-17

**Authors:** Haruhisa Konishi, Iemasa Koh, Noriyuki Shiroma, Yukie Kidani, Satoshi Urabe, Norifumi Tanaka, Eiji Hirata, Koji Arihiro, Yoshiki Kudo

**Affiliations:** ^1^Department of Obstetrics and Gynecology, Graduate School of Biomedical Science, Hiroshima University, Japan; ^2^Department of Anatomical Pathology, Hiroshima University Hospital, Japan

## Abstract

Intravenous leiomyomatosis (IVL) is a rare benign neoplasm. Herein, we describe two cases of IVL at different levels of progression. The tumor in Case 1 was extensive, invading the right atrium after a hysterectomy for a uterine myoma. The tumor temporarily responded to hormonal treatment; however, tumor regrowth occurred. In contrast, the tumor in Case 2 extended only to the pelvic veins and was revealed preoperatively. Hysterectomy and bilateral salpingo-oophorectomy were performed, resulting in the complete surgical resection of the tumor. In Case 2, no recurrence has been observed. Tumor samples were evaluated for hyaluronan expression using Alcian blue staining (with and without hyaluronidase digestion). The tumor in Case 1 stained strongly positive for hyaluronan while the tumor in Case 2 stained weakly positive for hyaluronan. In contrast, a large non-IVL uterine leiomyoma (control) stained negative for hyaluronan. These results suggest a relationship between tumor hyaluronan expression and IVL progression, similar to that in other cancers.

## 1. Introduction

Leiomyomas are the most common type of benign neoplasm in the uterus. Intravenous leiomyomatosis (IVL) is a rare variant of leiomyoma; IVL tumors grow within the uterine and extrauterine venous system. Although IVL tumors are histologically benign, IVL is potentially life-threatening, as the tumor can extend into the inferior vena cava (IVC), right cardiac chambers, and pulmonary arteries. Thus, patients with IVL can present with findings of hemodynamic compromise, such as dyspnea, syncope, congestive heart failure, or sudden death. Although IVL may be incidentally discovered early (in the uterine veins alone) when a patient undergoes a hysterectomy for reasons other than leiomyoma, most reports are of patients in the advanced stage of the disease, with right cardiac chamber or pulmonary artery involvement.

Hyaluronan is known to enhance tumor growth by promoting angiogenesis in various tumors [1, 2]. Herein, we report two cases of IVL at different levels of progression, concordant with different hyaluronan expression levels. Thus, these cases suggest a relationship between tumor hyaluronan expression and IVL progression.

## 2. Case Reports

### 2.1. Case 1

**Figure 1:**
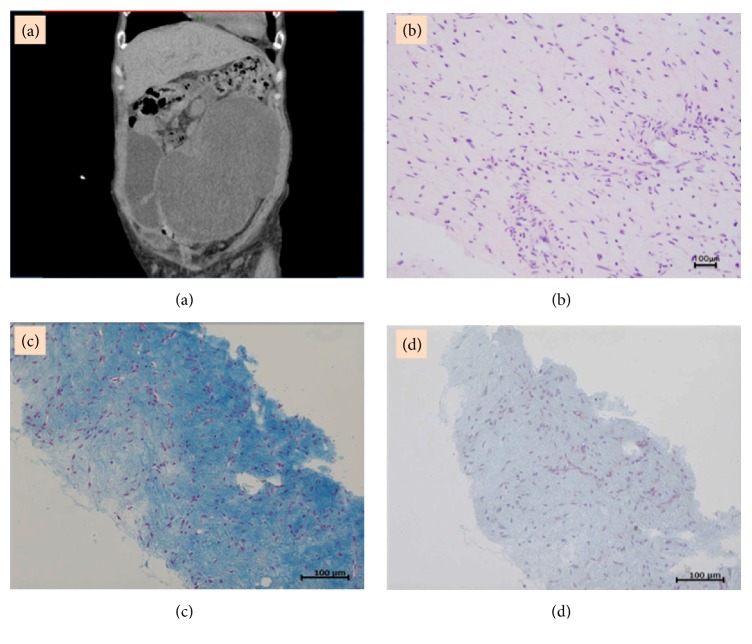
(a) Large tumor occupying the abdominal cavity on a computerized tomography scan. (b) The histopathological diagnosis is leiomyoma (H&E 200×), based on the absence of nuclear atypia and a low mitotic index. (c) Tumor cells stained strongly positive for Alcian blue at a pH of 2.5 (200×). (d) Alcian blue staining disappears after hyaluronidase digestion (200×).

A 55-year-old woman (gravida 1, para 1) was referred to our hospital because of the progression of a lower abdominal tumor. At 45 years of age, she underwent a total abdominal hysterectomy (TAH) at another hospital for a leiomyoma, which persisted after the surgery. One year later, an attempt to reduce the progressing residual tumor was unsuccessful. Two years after the TAH, the tumor had extended into the IVC and right cardiac chamber; thus, she underwent tumor resection surgery at another hospital and was admitted to our care some years after her last surgery. Computerized tomography (CT) revealed a large tumor occupying the abdominal cavity and multiple bilateral pulmonary nodules (Figure 1(a)). The patient's course was complicated by renal failure due to ureteric stenosis, secondary to the expanding tumor. Her serum estradiol level was 11 pg/ml and FSH level was 103 mIU.

A transabdominal needle biopsy was performed to exclude a malignant tumor; there was no nuclear atypia and the mitotic index was low. Thus, the final histopathological diagnosis was leiomyoma (Figure 1(b)). On immunohistochemistry, the tumor was positive for estrogen and progesterone receptors. In addition, the tumor cells stained strongly positive for Alcian blue (pH = 2.5). Moreover, the staining disappeared after hyaluronidase digestion, suggesting that the tumor contained abundant hyaluronan (Figures 1(c) and 1(d)). Thus, she was diagnosed with IVL and benign metastasizing leiomyoma.

The tumor temporarily responded to hormonal treatment (letrozole, medroxyprogesterone) and became smaller. However, the tumor eventually progressed. Among other conditions, she had a progressing lung metastasis, gastrointestinal obstruction, repeated cellulitis, and leg edema. The patient died of multiple organ failure due to tumor progression, 13 years after her initial surgery.

### 2.2. Case 2

**Figure 2:**
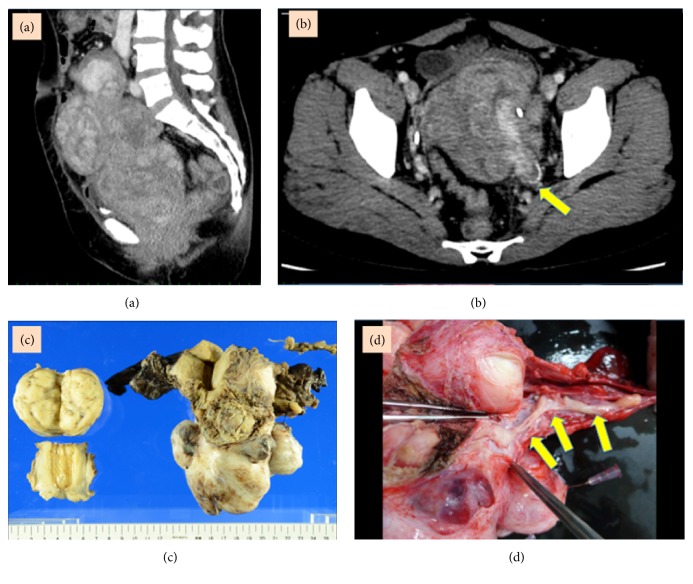
(a) Heterogeneous large tumor occupying the pelvic cavity on a computerized tomography scan. (b) Intravascular tumor within the dilated left internal iliac vein on a computerized tomography scan. (c) Multiple leiomyomas within the uterine corpus and cervix. (d) Tumor growth extending into the left ovarian vein.

A 46-year-old woman (gravida 2, para 2) was referred to our hospital complaining of a lower abdominal mass and pain. Her medical history was unremarkable. She was initially diagnosed with a uterine leiomyoma by transcervical needle biopsy. CT revealed a large heterogeneous tumor occupying the pelvic cavity and an intravascular tumor within the dilated left internal iliac and ovarian veins (Figures 2(a) and 2(b)). Her preoperative cervical cytology results were negative for intraepithelial lesions and malignancy. The endometrial cytology and needle biopsy results were also negative. Thus, the preoperative diagnosis was IVL, with extension of the tumor into the left internal iliac and ovarian veins.

Intraoperatively, multiple myomas were found within the uterine corpus and cervix, and the tumor extended to the parametrium and paracolpium. Detachment of the tumor from the left ureter and vaginal wall was very difficult. Intravenous tumors in the left internal iliac and ovarian veins could be palpated. The left internal iliac vein forming the common iliac vein was transected at the bifurcation region. In addition, TAH and bilateral salpingo-oophorectomy (BSO) were performed, resulting in the complete surgical resection of the tumor (operative time, 11 hours; blood loss, 8462 g). The resected uterus and adnexa weighed 897 g (Figures 2(c) and 2(d)). There was no residual tumor detected in the venous resection stump.

**Figure 3:**
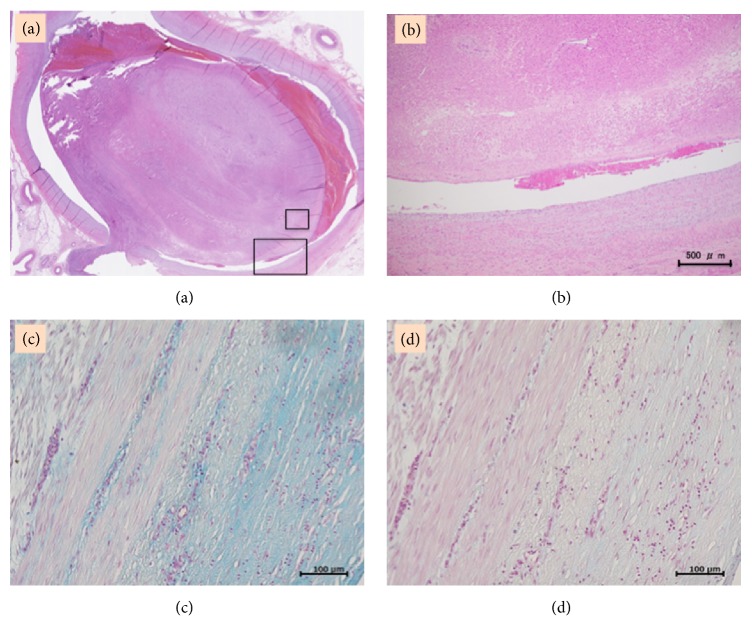
(a) The intravenous tumor (H&E magnifying lens). (b) Vessel endothelium cells and vascular smooth muscle layer covered the intravenous leiomyoma (H&E 40×); an enlarged view of the area in Figure 3(a) is surrounded by a large black box. (c) Tumor cells stained strongly for positive Alcian blue at a pH of 2.5 (200×); an enlarged view of the area in Figure 3(a) is surrounded by a small black box. (d) Alcian blue staining disappears after hyaluronidase digestion (200×); the same area as that in Figure 3(c) is shown.

The nodule resected from the uterus and the internal iliac and ovarian veins consisted of a proliferation of spindle cells. There was no nuclear atypia and the mitotic index was low. In addition, vessel endothelium cells and a vascular smooth muscle layer covered the IVL (Figures 3(a) and 3(b)). The tumor cells stained positive for Alcian blue (pH = 2.5) and the staining disappeared after hyaluronidase digestion. However, compared to that in Case 1, the intensity of the staining was weaker and less diffuse (Figures 3(c) and 3(d)). Similar findings for hyaluronan expression were obtained using the sample retrieved from the preoperative needle biopsy.

The histopathological diagnosis of the uterine and intravascular tumors was IVL. There has been no evidence of IVL recurrence, with the most recent follow-up at 38 months postoperatively.

## 3. Discussion

IVL was first described by Hirschfield in 1896 [3] and was defined by Norris and Parmly in 1975 [4]. IVL is histologically defined as a benign smooth muscle cell tumor; however, given their potential to grow within the uterine venous system, IVL tumors can cause a fatal cardiac obstruction. The early diagnosis of this condition is rare because of its low prevalence and nonspecific initial manifestations. Not infrequently, the diagnosis is made after death due to congestive heart failure. Differential diagnoses include leiomyosarcoma, endometrial stromal sarcoma, and diffuse uterine leiomyomatosis. The true rate of recurrence of completely resected IVL is unknown, but regrowth has been documented in up to 30% of patients. Thus, long-term follow-up imaging is recommended. CT appears to adequately detect the regrowth [5].

Although the precise pathogenesis of the intravenous invasion remains unclear, there are two theories regarding the origin of IVL tumors. The first suggests that the tumors arise from smooth muscle in the vessel wall, while the second considers IVL to be the consequence of a uterine leiomyoma invading into the surrounding vessels [6, 7]. Recently, Fukuyama et al. [8] reported that tumors advance by stretching the vascular wall (rather than by breaking the wall), progressing into the vein like a polyp, covered in endothelium cells. We are inclined to support the latter theory regarding the pathogenesis of IVL, as our pathological findings were of a tumor covered in endothelium cells (Figures 3(a) and 3(b)).

Controversy exists regarding the treatment of IVL. Given that IVL tumors are positive for estrogen and progesterone receptors, BSO is essential and exogenous estrogen must be avoided. Although hormonal treatments, such as gonadotropin releasing hormone (GnRH) analogs and antiestrogens, have been used to prevent tumor growth, evidence regarding their efficacy is inconsistent [4]. In Case 1, treatment with Letrozole (an aromatase inhibitor) and medroxyprogesterone resulted in a temporary response; however, the tumor eventually progressed. Currently, the complete resection of all existing and visible tumors is recommended, if possible [9]. However, an early-stage diagnosis is critical for a complete resection and good prognosis, which is rare, as the clinical presentation is initially nonspecific (e.g., pelvic pain and abdominal discomfort). In Case 2, we suspected IVL because of the enlarged veins and intravenous tumors on CT imaging. This preoperative diagnosis enabled the necessary preparations for a complete resection.

Hyaluronan is a component of the extracellular matrix and is involved in various aspects of mammalian tissue physiology. Hyaluronan binds to CD44 receptors, mediating many cellular events, including cellular regeneration and wound healing. However, abnormalities in hyaluronan production, resulting in Rho A and PI3K Rac cascade activation, have been implicated in many diseases, including cancer [1, 2]. In addition, high levels of hyaluronan expression have been observed in IVL tumors [10, 11]. We noted that hyaluronan was expressed in the IVL tumors in both of our cases. Furthermore, the tumor in Case 1 had a higher level of hyaluronan expression and more progressed IVL compared to that in Case 2. In contrast, hyaluronan expression in a large leiomyoma was negative (data not shown). Thus, increased hyaluronan expression in IVL tumors may indicate that the tumors are highly angiogenic and have a potential for invasion. Consistent with this, a close relationship between tumor hyaluronan expression and the progression of breast and colorectal cancers has been demonstrated [12, 13].

Alcian blue staining was used to evaluate the hyaluronan expression level in IVL tumors. Alcian blue staining is a common and well-validated staining technique that is used to detect acid mucopolysaccharides, including chondroitin sulfate and hyaluronan. Hyaluronan can be distinguished from other materials by the disappearance of the stain after hyaluronidase digestion. Myxoid leiomyomas and myxoid leiomyosarcoma have also been reported to stain positive with Alcian blue [14].

In this report, we described two cases of IVL with different outcomes. The accumulation of additional cases is needed to further evaluate a relationship between hyaluronan expression, assessed with Alcian blue staining, and IVL progression.

## Data Availability

The datasets analyzed during the current study are available from the corresponding author upon reasonable request.
